# High-throughput and Cost-effective Chicken Genotyping Using Next-Generation Sequencing

**DOI:** 10.1038/srep26929

**Published:** 2016-05-25

**Authors:** Fábio Pértille, Carlos Guerrero-Bosagna, Vinicius Henrique da Silva, Clarissa Boschiero, José de Ribamar da Silva Nunes, Mônica Corrêa Ledur, Per Jensen, Luiz Lehmann Coutinho

**Affiliations:** 1 Animal Biotechnology Laboratory, Animal Science and Pastures Department, University of São Paulo (USP)/Luiz de Queiroz College of Agriculture (ESALQ), Piracicaba, São Paulo, Brazil; 2 IFM Biology, AVIAN Behavioural Genomics and Physiology Group, Linköping University, Linköping, Sweden; 3 Brazilian Agricultural Research Corporation (EMBRAPA) Swine & Poultry, Concórdia, Santa Catarina, Brazil

## Abstract

Chicken genotyping is becoming common practice in conventional animal breeding improvement. Despite the power of high-throughput methods for genotyping, their high cost limits large scale use in animal breeding and selection. In the present paper we optimized the CornellGBS, an efficient and cost-effective genotyping by sequence approach developed in plants, for its application in chickens. Here we describe the successful genotyping of a large number of chickens (462) using CornellGBS approach. Genomic DNA was cleaved with the *PstI* enzyme, ligated to adapters with barcodes identifying individual animals, and then sequenced on Illumina platform. After filtering parameters were applied, 134,528 SNPs were identified in our experimental population of chickens. Of these SNPs, 67,096 had a minimum taxon call rate of 90% and were considered ‘unique tags’. Interestingly, 20.7% of these unique tags have not been previously reported in the dbSNP. Moreover, 92.6% of these SNPs were concordant with a previous *Whole Chicken-genome re-sequencing* dataset used for validation purposes. The application of CornellGBS in chickens showed high performance to infer SNPs, particularly in exonic regions and microchromosomes. This approach represents a cost-effective (~US$50/sample) and powerful alternative to current genotyping methods, which has the potential to improve whole-genome selection (WGS), and genome-wide association studies (GWAS) in chicken production.

Next-generation sequencing (NGS) analyses have been increasingly employed in production animals, particularly in chickens. NGS generates large amounts of genomic information that can be used to detect genetic variants related to functional alterations^1^. Single Nucleotide polymorphisms (SNPs) are the most abundant type of molecular markers and their high genomic density facilitates their interrogation by different genetic approaches. These include large-scale genome association analyses, genetic analysis of simple and complex disease states, and population genetic studies^2^.

The use of NGS has enabled to identify SNPs across genomes and allowed the development of pre-designed SNP chips for widespread testing of SNP associations with specific phenotypes of interest^3^. However, pre-designed SNP chips have limited coverage on functionally important genomic regions in experimental populations. SNP chips generally contain a limited number of SNPs in coding or regulatory regions, rarely contain SNPs with significant effects^4^, and include non-polymorphic SNPs, which difficults tracking their inheritance in specific pedigrees^5^. On the other hand, although NGS has enough power to detect informative polymorphisms, its high cost makes its use impractical in animal breeding and genome-wide selection^6^,^7^.

The use of an economical, efficient, and simple-step method of SNPs discovery, validation and characterization that uses reduced representation sequencing generated by restriction enzyme cleavage of target genomes can provide an unbiased genome-wide set of SNP markers in different genomes^7^, including chickens^8^. Reduced representation methods can be grouped in three classes: (1) reduced-representation sequencing, which includes methods such as reduced-representation libraries (RRLs) and complexity reduction of polymorphic sequences (CRoPS); (2) restriction-site-associated DNA sequencing (RAD-Seq); and (3) low coverage genotyping, which includes methods such as multiplexed shotgun genotyping (MSG), genotyping by sequencing from Cornell (CornellGBS)^9^, and genome reducing and sequencing (GGRS)^10^. Of these reduced representation methods, RAD-Seq^8^ and GGRS^10^ have been employed in chickens^9^. The possibility to reduce the genome complexity using restriction enzymes that generate DNA fragments of specific ranges^11^ expedite re-sampling and produces coverage levels that are acceptable for solid SNP calling^12^.

CornellGBS is a simple reproducible method based on the Illumina sequencing platform^13^ that requires low input of DNA (100 ng). This method allows for a highly multiplexed approach, which is achieved through the incorporation of unique barcodes that identify individual samples in a DNA pool to be sequenced. This approach avoids the low sequence diversity in which the restriction enzyme overhangs appear at the same position in every read, by employing barcodes of variable lengths^9^. In addition to the methodological simplicity of simultaneously discovering and characterizing polymorphisms, the availability of an open-source analysis tool is a major advantage of the CornellGBS approach^13^. This methodology is currently being successfully applied in numerous species by a large number of researchers^7^. However, to the best of our knowledge this method has not been applied in chicken.

The present study aims at constructing reduced genome representation sequencing libraries using the CornellGBS approach in chickens. In order to optimize the use of CornellGBS in chickens, cleavage of the chicken genome was tested with two different restriction enzymes, *PstI* and *SbfI*. Two different experimental animal populations were used in the present study: 444 chickens from five families of the EMBRAPA F_2_ Chicken Resource Population (Concórdia, SC, Brazil), 8 chickens from the F_1_ generation and 18 chickens from the parental line (F0). In the present article, we have optimized the use of CornellGBS in chickens, which was achieved in part by using the *Pst1* restriction enzyme for genomic cleavage. We also provide a new set of chicken SNPs that were detected by using this approach. The application of this methodology will open many possibilities for downstream applications in chickens and facilitate SNP discovery in specific populations of chickens. The relevance of applying a cost-effective genotyping method in chickens is enormous, given their world-wide economic relevance as production animal^14^.

## Results

### Enzyme selection and library fragment size distribution

The selection of the appropriate restriction enzyme was based on relevant literature information and took into consideration the number of expected fragments, the fraction of the diploid genome sampled, and the expected number of reads required to obtain a sequencing coverage of ~7X of sequencing coverage^15^. The library complexity depends on the relation between the enzyme selected and the species’ genome under investigation. Therefore, the level of DNA methylation sensitivity and recognition site size in relation to the genome under study had to be tested^16^.

**Figure 1:**
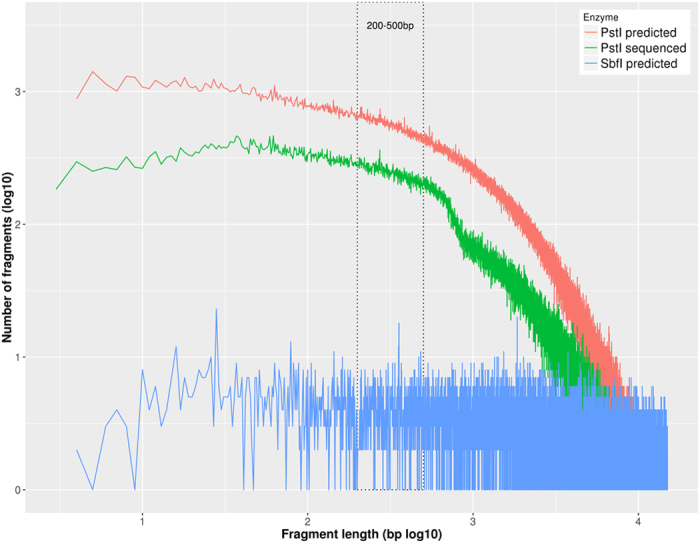
Comparison of patterns of genomic cleavage using *PstI* or *SbfI* restriction enzymes. For cleavage with *PstI* both the predicted (*in silico*) and the obtained pattern after sequencing are shown. Only the predicted (*is silico*) pattern of cleavage is shown for *SbfI* since the pattern generated did not satisfy the requirements for being used in the CornellGBS. The region framed with dashed lines contain fragments in the 200–500 bp length range, which is the range of interest for further Illumina sequencing.

We initially selected two enzymes that are insensitive to *dam, dcm* and *CpG methylation* according to the manufacturer (NEB BioLabs). These enzymes were *PstI* and *SbfI. In silico* cleavage of the chicken genome was performed with both PstI and *SbfI* enzymes. Genome cleavage with *PstI* generated 811,951 fragments, while *SbfI* generated 45,116 fragments. Fragment size distribution obtained with *PstI* showed a low amount of discreet size, which is indicative of low repetitive fragments^13^ (Fig. 1). Each enzyme generated a different distribution of fragment lengths across the entire genome.

Fragments ranging between 200–500 bp were generated and mapped against chromosome locations in the chicken genome (galGal 4; GGA). Pst1 *in silico* digestion generated 159,673 fragments, which were evenly distributed across all the chromosomes, while *SbfI* cleavage generated 1,186 fragments (Supplementary Fig. S1). There is a tendency with the *PstI* cleavage to generate clusters with similar range of fragment lengths, particularly between 200 and 500 bp, which is an appropriate length for sequencing by the HiSeq Illumina platform^17^. Cleavage with *SbfI*, however, generated fragments of a variety of sizes and in lower quantity compared to *PstI* (134.6 times less then *PstI* cleavage) (Fig. 1). Importantly, 40% of the fragments generated by *Sbfl* are outside the range showed on Fig. 1, representing fragments larger than 15 Mbps.

We also performed a comparison between the mapping of fragments (*tags*) generated by the *in silico* cleavage (*Predicted PstI-Tags*) and the *tags* generated after the *in vitro* cleavage (*Sequenced Pst1-Tags*) of 462 individuals (Fig. 1). In both cases the tags were aligned against the chicken reference genome (*Gallus gallus 4.0*, NCBI). The number of Predicted *PstI*-Tags obtained was 811,951, while the Sequenced *PstI*-Tags obtained were 287,819. Detailed information on the size categories of the Predicted *PstI*-Tags that were actually sequenced is provided in Supplementary Table S1.

Agarose gel electrophoresis of the chicken genomic DNA digested with the *PstI* and *SbfI* restriction enzymes revealed a more efficient cleavage with *PstI* (see Supplementary Fig. S2).

### Sequencing and alignment

**Figure 2:**
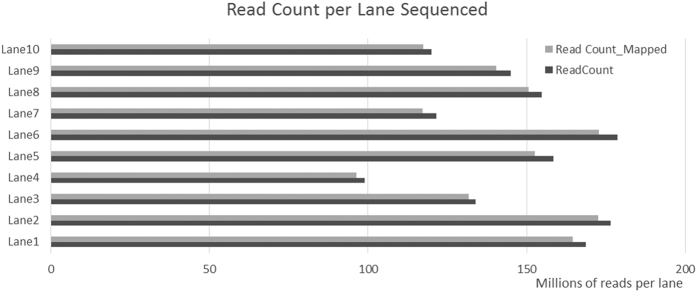
Distribution of the number of sequenced reads counted and mapped per flowcell lane.

The 48-plex *Pst*I-digested libraries were run in 10 lanes of Illumina flow cells. Approximately 3.6 × 10^9^ short reads (100 bp) were generated. After quality trimming by the SeqyClean tool^18^ approximately 1.8 × 10^9^ reads (52%) were retained. A high number of short fragments (<50 bp) sequenced were eliminated in the fragment size filtering (37%), as well as contaminants (11%). Approximately 1.4 billion reads were retained after application of the *Tassel* filter (*reads* >64 bp and properly identified with barcodes). These reads were distributed at an average of 145.6 (±26.5) million reads per lane (Fig. 2). These results represent 3.1 (±1.7) million reads per individual chicken, of which 3.0 (±1.7) million reads were successfully mapped (plots including read count per animal are provided in Supplementary Fig. S4).

The number of unique sequence tags (from 464 individuals altogether) that aligned against the chicken reference genome (*Gallus gallus 4.0*, NCBI) was ~5.4 million and 92.8% of them could be mapped. The average sequencing coverage depth was ~264 reads per tag (locus) in these ~5.4 million unique tags.

These ~5.4 million unique sequence tags represent a 4.66% coverage of the whole chicken-genome (~50 million bp). The average coverage for the 464 chickens was 5.6 X for the targeted regions.

### SNP discovery

**Figure 3:**
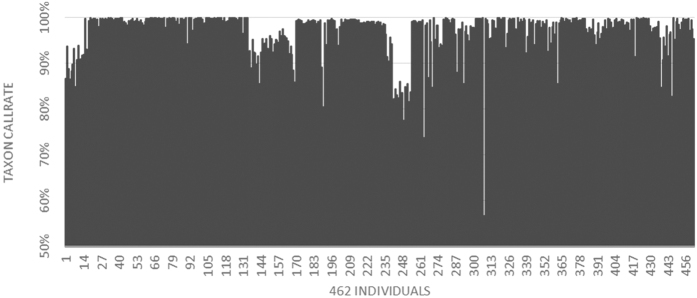
Distribution of the 462 taxon call rates representing the percentage of total SNPs called. The x-axis represents the 462 individuals (taxon) and the y-axis represents the taxon call rate.

From these ~5.4 million unique sequence tags, 327,240 SNPs were identified considering a minimum minor allele frequency (mnMAF) of 1%. Two of the 464 individuals showed a minimum taxon coverage (mnTCov) of less than 20% and were eliminated from the analysis. The minimum site coverage (mnScov) filter was used to evaluate the taxon call rate. The number of remaining *PstI*-derived SNPs was 134,528 after applying a mnScov filter of 70%, and 67,096 SNPs after applying a mnScov filter of 90%. After filtering with a mnScov of 90% the average taxon call rate per individual was 97% (Fig. 3).

Additionally, we also tested a mnMAF of 0.05, which generated 300,777 SNPs, as well as a combination of mnTCov of 20% and mnScov of 90%, which generated 61,618 SNPs.

### Comparison of genotyping methods and CornellGBS validation

**Figure 4:**
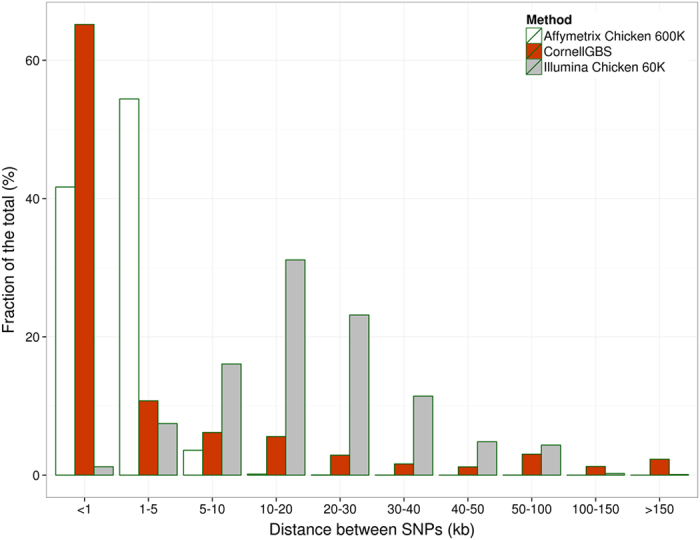
Distribution of distance ranges between SNPs. SNPs were mapped against chromosome locations of the chicken genome after being detected with Affymetrix 600K, CornellGBS or Illumina Chicken 60K Beadchip. The *x*-axis represents the distances between adjacent SNPs (Kb) and the *y*-axis represents the fraction of the total SNPs called.

When considering a mnMAF of 1% and mnScov filter of 90% the *PstI*-derived SNPs are shown to be separated by a distance of 15 Kb in average, with a median of 55 bp. This indicates clusters of SNPs in regions represented by the cleaved areas (tags). A comparison among the different genotyping methods is shown on Table 1, Fig. 4 and Supplementary Table S4. The distances between SNPs ranged between 1 bp–1.8 Mb (Table 1), and the majority of the SNPs were separated by distances <1 Kb (Fig. 4).

Differences were found between SNP numbers and density (SNP/Mbp) inferred by the three methods used for SNP detection, namely Affymetrix 600 K, Cornell GBS and Illumina chicken 60K bead chip (Supplementary Table S4). In order to test for differential representation of the SNPs obtained across the chromosomes, the chicken genome was divided into three categories: large chromosomes (GGA1-5, Z), corresponding to ~68% of the chicken genome; medium-size chromosomes (GGA6-10) corresponding to 15% of the chicken genome, and microchromosomes corresponding to 17% of the chicken genome^19^. The representation of SNPs in each chromosomal category is shown in Table 2.

The set of 67,096 SNP chromosomal positions obtained with the CornellGBS (mnMAF 1% and mnScov 90%) was compared to the 12,357,602 filtered SNPs from a *Whole Chicken-genome re-sequencing* ~11X (WCGR) dataset (Boschiero *et al.*, unpublished results) in order to perform a validation of the method since both sets were obtained from the same 10 animals (TT and CC lines). The SNPs with more than one alternative allele (less frequent) were eliminated from this analysis. A concordance of 83.91% (49,680) in the chromosomal positions of the SNPs detected was observed between the two methods. We found that 92.64% of these concordant markers had concordant genotypes between CornellGBS and WCGR datasets. Also, the consistency in the calls of heterozygosity was tested between these two approaches. This test was performed due to the general assumption that reduced representation methods, like CornellGBS, have limitations in the calling of heterozygous SNPs^7^. It was observed that 71.32% of all heterozygous SNPs evaluated here (149,741 genotype comparisons) were validated against the WCGR dataset. However, 86.88% of the non-concordant genotypes occurred because the CornellGBS considered the genotype as homozygotic, and WCGR as heterozygtic. In addition, we found that when both methodologies were able to call heterozygous (106,906 genotype comparisons), 99.90% of the genotypes were in agreement. Interestingly, the number of heterozygous calls in the region assessed was similar between the CornellGBS (112,435) and the WCGR (144,112) approaches, corresponding to 24.15% and 29.18%, respectively, of all the genotype comparisons.

### Homozygous and heterozygous genetic variants

Out of 31 million possible genotypes (462 taxon × 67,096 sites), the proportion of heterozygous SNPs was 31%, with 3.1% being missing data (see Supplementary Table S2). The average heterozygosity observed ranged between 9.7–48.5%, with 18% of coefficient of variation (CV).

A lower proportion of heterozygous SNPs was found in both parental lines CC (0.20 ± 0.01) and TT (0.26 ± 0.01), followed by the F_2_ (0.31 ±  0.05) and the F_1_ generations (0.32 ± 0.10) (Table 3). The F_1_ generation had the highest CV due to the fact that it represents a heterozygous population. The family F_2_-7816 had a higher CV (25%) compared with the other F_2_ families due to the low heterozygous call rate for some individuals (25 from 94) in this family.

### Functional Annotation

**Figure 5:**
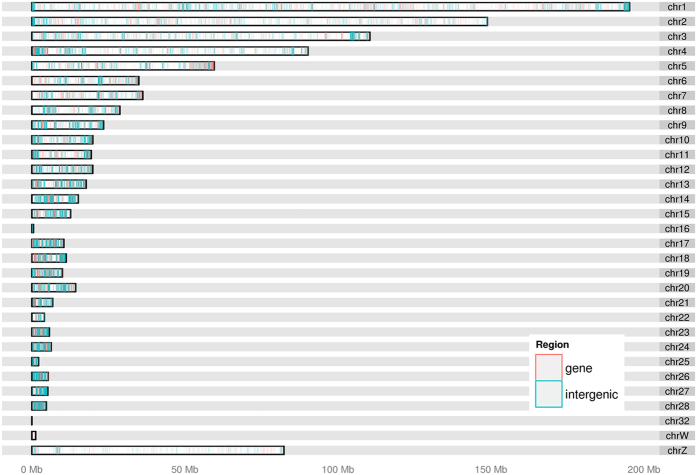
Karyotype of the SNP distribution in genic (red) and intergenic (blue) regions of the *Gallus gallus* genome. The *x*-axis represents the chromosome size (Mbp). The *y*-axis represents the chromosomes.

The unique set of 67,096 *PstI*-derived SNPs (after filtering) from the 462 chickens were annotated against the known genes from the ENSEMBL database (see the graphical representation of SNPs distributed in genic and intergenic regions of the chicken genome on Fig. 5). Among the variants found, 20.7% (13,918) were new, while 79.3% (53,178) were already described. Functional annotation of these novel SNPs was performed using the chromosomal positions of the most recent update of chicken genome (*Gallus gallus 4.0*, NCBI) as a reference. The results are available in the supplementary materials (Supplementary Spreadsheet S1).

From these 67,096 *PstI*-derived SNPs, 11,372 SNPs had multiple annotations (totalizing 78,399 annotations) as they could be considered into multiple variant classifications (Table 4). The non-synonymous SNPs were analyzed by the SIFT algorithm, which predicts whether genetic variants can affect protein function. This is performed by assessing the level of conservation in homologous protein sequences^20^. The program predicted the SIFT score for 650 SNPs from the 907 non-synonymous SNPs. From these 650 SNPs, 155 SNPs (23.8%) were non-tolerated variants (SIFT score ≤0.05) (see Supplementary Table S3).

### Mendelian inheritance of the SNPs detected

In addition to the SNP validation we also tested for Mendelian errors in the markers obtained in each population used in this study. This test was performed in the complete dataset of 67,096 *PstI*-derived SNPs, as well as in the subset of 13,543 novel SNPs. The results are shown in Table 5.

### Genetic map construction

We performed a linkage analysis in which the SNPs were tested against the expected segregation ratio. Three genotype combinations in the parental lines were informative for the construction of a genetic map: two combinations when one parent was heterozygous and the other was homozygous (AA × AB or AB × AA) and one combination when both parents were heterozygous (AB × AB). The SNPs following each of these segregation patterns in the parents were retained and markers with significant segregation distortion (P < 0.001, χ2 test) were removed from the map construction. A total of 6,037 SNPs were retained for linkage map construction after filtering, with 387 of these SNPs being classified as female heterozygous, 2,143 SNPs classified as male heterozygous, and 3,507 SNPs classified as heterozygous in both genders.

**Figure 6:**
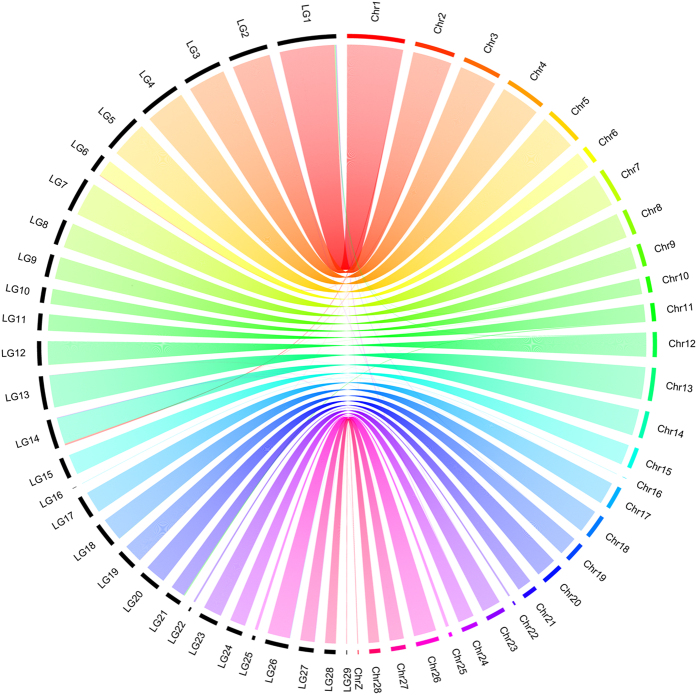
Whole-genome synteny between the physical maps obtained from 5,842 *PstI*-derived SNPs that formed non-fragmented LGs. Each line represents a connection between the chromosomal placement of a particular marker in our linkage map (black; scale in cM) and a homologous sequence in the physical map (non-black colors; scale in Mb).

From the retained 6,037 *PstI*-derived SNPs, 5,982 generated 53 linkage groups (LG) that corresponded to the chromosomes GGA1-28 and Z (see Supplementary Fig. S5). We had no informative markers for chromosomes GGA32 and GGAW LGs. From these 5,982 SNPs that originated LGs, 5,842 markers formed 29 non-fragmented LGs, i.e. markers in agreement with their respective described chromosomes (shown in the physical map, Fig. 6). Of the remaining markers, 140 formed fragmented LGs, while 55 were considered unlinked. Within these 29 LGs originated, 73 markers were in disagreement with their respective LGs (Fig. 6).

## Discussion

CornellGBS is a widely employed method for genotyping large genome species because it is simple, fast, specific, reproducible, and interrogates important regions of the genome that are inaccessible to sequence capture approaches^21^. Although this methodology was first reported in maize^6^, its application was recently expanded to bovine^13^. Moreover, a similar technique called GGRS was recently applied in chickens^10^. In the present study we have adapted the CornellGBS successfully to be applied in chickens using a restriction enzyme that generates an appropriate genomic shearing range for this species. This work describes for the first time the application of the CornellGBS method for chicken genotyping. This is a cost-effective genotyping method that was performed here in a large number of individuals (462 chickens).

The GBS approach involves four steps: (1) genomic DNA cleavage, (2) adapter ligation with specific barcodes, (3) sequencing of short reads, and (4) bioinformatics analysis.

The first step in the method adaptation for its use in chickens is the selection of an appropriate restriction enzyme to shear the chicken genome in a suitable range of fragments for sequencing by the Illumina platform. We performed *in silico* (Fig. 1) and *in vitro* (see Supplementary Fig. S2) genomic fragmentation tests to compare the digestion profiles of two restriction enzymes, *PstI* and *SbfI.*

The CornellGBS approach is flexible enough to be applied on different genomes. However, the choice of a restriction enzyme that cleaves the DNA generating a suitable fragment length range is of particular importance. Moreover, genomes of different species will produce distinct cleavage patterns with the same enzyme, reason why optimization is required for the genomic cleavage in each species^7^. It is also important to consider whether the restriction enzyme is sensitive to DNA methylation in its restriction site^16^,^22^. *Pst1* showed here the best fragmentation profile among the two enzymes tested for cleaving the chicken genome, both *in vitro* and *in silico*. The next step after the selection of the appropriate restriction enzyme was to optimize the binding reactions between the fragments, adapters and barcodes.

After sequencing of the CornellGBS libraries the next step was the bioinformatics analysis. Using the Tassel pipeline ~5.3 million of unique tags were obtained and aligned against the last chicken reference genome (*Gallus_gallus_*4.0, NCBI). Although 48% of the reads were discarded, which can be considered a drawback of CornellGBS approach^10^, the number of unique tags obtained (1.4 billion) is sufficient for an accurate identification of SNPs. As a matter of comparison, a similar study generated ~0.5 million unique tags using 47 individuals^13^. The multiplexing capability is an advantage of the CornellGBS approach that increases the catalog size of unique tags.

Most of genotyping methods have limitations when it comes to detection of heterozygous SNPs, due to the low coverage of these sites^17^. For a coverage of less than 5X per site per individual the probability that only one of the two chromosomes of a diploid individual is sampled for a particular site is generally high^23^. The tassel-GBS pipeline compensates low coverage data and under-calling of heterozygotes with the redundant coverage of haplotypes at high marker density, which facilitates imputation of missing genotypes^7^. This is possible because in the Tassel-GBS pipeline the tag catalog is created from individuals pooled altogether, rather than from separate individuals. The latter is the case for the Stacks software, a program commonly used to handle GBS data^24^.

Different filtering parameters on SNP calling were tested in the present study. Since using a mnMAF of 5% (Tassel default) generated 5,478 less SNPs than using a mnMAF of 1%, we proceeded with a mnMAF of 1%. Moreover, because parental pure lines featuring only 5 individuals per strain were used, the previous mnMAF ≥5% would eliminate many important SNPs that might be present in the parental lines.

When considering a mnMAF of 1% and mnScov filter of 90%, our study generated a reliable SNP dataset of 67,096 *PstI*-derived SNPs, out of which 20.7% have not been previously described in the dbSNP database (based on the last update of the dbSNP database, NCBI, September 2015). A previous study that used RAD-Seq in chickens^8^ found 28,895 *HindIII*-derived SNPs candidates with 53.3% of them newly reported (based on a previous version of dbSNP database, which contains fewer SNPs). Therefore, a reasonable number of novel SNPs were obtained here using the CornellGBS approach (13,434).

In spite of the different SNP calling methodologies used (Stacks vs Tassel), the number of *PstI*-derived SNPs reported here was higher than *HindIII*-derived SNPs previously reported^8^. This is probably explained by the difference in the number of ‘tag counts’ observed after cleavage (*in silico)* by *HindIII* (~700 K, as previously reported^8^) and *PstI* (~1.2 million, reported here), or by the larger number of genotyped animals in the present study.

The chromosomal position of the SNPs identified in this study (using CornellGBS and considering a mnMAF of 1% and mnScov filter of 90%) was compared with the Illumina chicken 60K Beadchip^25^ and with the 600 K HD Affymetrix^®^Axion^®^ genotyping array for chicken^3^. We found that the average distances between markers obtained using the CornellGBS or the 60 K approaches were similar (15 and 21 Kb, respectively), although lower than with the 600 K (1.7 Kb), which showed less distance between markers.

Differences between mean and median were detected only using the CornellGBS approach. This indicates that SNP cluster formation occurs in spite of the markers obtained by the CornellGBS being well spread throughout the genome (Fig. 5). With the 60 K or the 600 K panel, however, uniform SNP distribution occurs without cluster formation. Also, differences between mean and median are not observed (Table 1). The detection of SNP clusters by the Cornell GBS approach lead us to perform functional annotation of the markers and compare the results between the methodologies tested. When the distribution of SNP distances was evaluated (Fig. 4) we noticed that the GBS and 600 K approaches had a similar proportion of SNPs that corresponded to the fraction of <1 Kb SNP distance between markers. Within GBS clusters the SNP density was higher and approximately 76% of SNPs were <5 Kb apart.

We also investigated SNP density differences related to chromosome size (see Supplementary Table S4) using the three methodologies (CornellGBS, 60K Illumina and 600K Affymetrix). CornellGBS detected about one-third more SNPs than the other two methods in regions of the microchromosomes GGA11-32 and W. The microchromosome GGA16 showed a higher representation of SNPs using the CornellGBS approach compared to the 60 K panel (0.19–0.05%). The GGAW microchromosome in the CornellGBS approach had two-thirds of SNP representation compared to the 600 K Illumina panel. Interestingly, SNPs in this microchromosome are not detected by the 60 K panel. SNPs in the GGA32 microchromosome were detected only by the CornellGBS approach. Interestingly, microchromosomes have 2–4 times higher gene density than macrochromosomes^19^,^26^ and ~48% of genes in microchromosomes have a high CpG island content^19^,^26^,^27^,^28^,^29^. This suggests *PstI* RE genomic cleavage would be appropriated for DNA methylation profiling, since it apparently enriches for regions of high CpG content.

A set of SNPs from the CornellGBS dataset obtained in our study was compared with a WCGR SNPs dataset (Boschiero *et al.*, unpublished results) obtained from sequencing the same 10 animals. Substantial chromosomal position (~84%) and genotype (~93%) concordances were observed between the two methods. However, the concordance was reduced to ~71% when considering only the heterozygous SNPs. In spite of this, 99.90% of the genotypes were concordant in regions where both methodologies were able to call heterozygous. Therefore, although the CornellGBS had fewer calls of heterozygous in comparison with WCGR, those genotypes that are called are quite reliable.

We also tested for Mendelian errors in the markers obtained in each population used in this study. Mendelian inheritance errors are likely to result from erroneous genotype calls^30^. The errors found were <10% between the parental (F_0_) and the F_1_ generation, and the same between the F_1_ and the F_2_ generations. The exception is, family F_2_-7816 that presented slightly higher Mendelian errors (11.9%). These error rates are in agreement with the low heterozygous call rate (0.90> call rate >0.95) and high heterozygous CV (>15%) observed in individuals from this family (25 from 94) when compared to the others four families (0.95> call rate >1.0). Therefore, the Mendelian errors observed were minimal and do not compromise the quality of the genotyping performed in the present study. In addition, the linkage map obtained from markers with Mendelian segregation obtained from the five F_2_ families were grouped in LGs. This grouping generated a fairly dense linkage map. These markers (~99% of them) grouped according to their respective described chromosomes (Fig. 6 and Supplementary Fig. S5).

We also found a small increase in the proportion of SNPs (3.3%) in exonic regions compared to a recent functional classification of 15 million SNPs detected from diverse chicken populations (2.2%)^1^, or when compared to the WCGR. These newly discovered SNPs in exonic regions include a QTL region on chromosome 3 associated with fatness in chickens (0.98%)^31^ and another on chromosome 2 associated with muscle deposition (0.59%)^32^. These *exonic* variants (2,590) were classified into functional categories due to their potential to alter the tri-dimensional structure and function of the translated protein^33^. These exonic variants detected in the present study were classified as non-synonymous, startlost, startgain or stopgain (Table 4).

When comparing the CornellGBS and the 60 K Illumina approaches (which have similar SNP density), it was observed that 60 K Illumina detects half (51.6%) of the exonic variants detected by CornellGBS. However, that difference is reduced when only non-synonymous SNPs are considered (907 SNPs detected by CornellGBS; 888 SNPs detected by 60 K Illumina). When comparing CornellGBS and 600 K Illumina, the proportions of SNPs in exonic regions are similar (3.3% and 3.5%, respectively). This shows that Cornell GBS is as powerful as the 600K panel in detecting SNPs in exonic regions, which is remarkable considering that the 600K panel was designed prioritizing coding regions^3^.

On the downside, CornellGBS seems to be less powerful in detecting SNPs in intergenic regions compared to either the 60 K Illumina or the 600 K Affymetrix approaches (28.21%, 43.68% and 41.77% respectively). On the other hand, CornellGBS presents a high proportion of SNPs in regions 1kb up- or downstream from UTR compared to either the 60 K Illumina or the 600 K Affymetrix approaches (14.69 and 15.70; 7.94 and 7.54; 7.58, 7.28; respectively). This is interesting because UTR regions are highly relevant for transcriptional regulation^34^.

These results indicate that the Cornell GBS approach shows a pattern of SNP profiling that is unique in comparison with other approaches. The unique characteristics of Cornell GBS include better interrogation of specific functional regions, of microchromosomes and of CpG-rich regions compared to other methodologies (60K Illumina or 600K Affymetrix). In particular, we believe that the restriction enzyme used in the present study (*PstI*) is responsible for enriching the cleaved genome for microchromosomic or CpG-rich regions.

The present study shows for the first time the application of CornellGBS in chickens, which will allow for the use of a cost-effective (~US$50/sample) genotyping approach in poultry. The method described is capable of performing a reliable SNP profiling in chickens using a large number of animals. In the present study a number of SNPs were discovered, which were well spread throughout all the chromosomes of the chicken genome (Fig. 5). This study describes a highly multiplexed sequencing method in chicken, with potential for application in studies involving genome-wide association and genomic selection.

## Methods

### Ethical statement

All experimental protocols employed in the present study that relate to animal experimentation were performed in accordance with the resolution number 010/2012 approved by the Embrapa Swine and Poultry Ethics Committee on Animal Utilization, in order to ensure compliance with international guidelines for animal welfare.

### Sample selection and preparation

This study was conducted using 464 chickens from an experimental population originated and maintained at the dependencies of the Brazilian Agricultural Research Agency, from (EMBRAPA; Concórdia, SC, Brazil). The population includes 446 chickens from five F_2_ families of the EMBRAPA F_2_ Chicken Resource Population, 10 chickens from their parental lines (5 from each line), and 8 chickens from the F_1_ generation.

The F_1_ generation individuals were originated from a cross between a parental broiler line (TT) and a layer line (CC), both developed at EMBRAPA. To generate the F_2_ population (TCTC), one F_1_ male (TC) and three F_1_ females (TC) were selected from different F_1_ families and were randomly mated with non-related animals. A more detailed description of the population has been previously provided^35^,^36^.

Genomic DNA was extracted from blood samples following proteinase K digestion (Promega), DNA precipitation in absolute ethanol, DNA washing in 70% ethanol and resuspension in ultrapure water. DNA samples were quantified in a fluorometer (Qubit^®^ Fluorometric Quantitation). Sample quality was assessed using the Nanodrop^®^2000c spectrophotometer and DNA integrity was checked in 1% agarose gel.

### Restriction enzymes selection and adapters design

*In silico* cleavage of DNA with *PstI* and *SbfI* was performed in R using the following Bioconductor^37^ packages: *Biostrings, BSgenome.Ggallus.UCSC.galGa14, plyr*, *ggplot2*, *reshape2* and *scales* (https://github.com/) (see Supplementary Fig. S1). The *in silico* cleavage was used to generate a dataset of fragments mapped against the galGal4 genome. The dataset of fragments that are predicted to be generated after *in silico* genomic cleavage with *PstI* was named ‘Predicted *PstI-Tags’*. The dataset of fragments that are predicted to be generated after *in silico* genomic cleavage with *SbfI* was named ‘*Predicted SbfI-Tags’*. The dataset of fragments that were obtained from the *in vitro* cleavage of the DNA from the all the 462 individuals analyzed was named ‘Sequenced *PstI-Tag’* and was generated using sam2bed from BEDOPS v2.4.15 tool. All the fragments either from *in silico* or *in vitro* analyses were aligned against the chicken reference genome (*Gallus gallus 4.0*, NCBI).

We also performed *in vitro* genomic cleavage of chicken DNA samples with the abovementioned restriction enzymes (see Supplementary Fig. S2), according to the New England BioLabs^®^ manufacturer´s protocol.

The adapters were designed using the GBS Barcode Generator tool (Deena Bioinformatics) taking into consideration the barcode sequence, in order to maximize the balance of the bases at each position in the defined set^6^.

### Preparation of sequencing libraries

After *PstI* digestion, adapters were linked to the cohesive ends of the digested DNA with T4 DNA ligase (New England BioLabs^®^). Approximately 24 samples were polled and purified using *QIAquick PCR Purification Kit*^*®*^(Quiagen). The fragments of each library were amplified by PCR using specific primers for sequencing in the Illumina platform. The purification of PCR reactions was performed using the *Agencourt AMPure XP PCR purification kit*^*®*^(Beckman Coulter) (see Supplementary Fig. S3). Each library was quantified by quantitative PCR using the *KAPA Library Quantification Kit* (KAPA Biosystems). Two pools of ~24 samples containing equal concentration of DNA were sequenced per flowcell lane totaling ~48 samples sequenced with different barcodes in each flowcell lane. Sequencing libraries were diluted to 16 pM and clustered using the cBOT (Illumina) equipment. Paired-end sequencing with a read length of 100 bp was performed using the HiSeq2500 instrument from Illumina. For the analysis we used the HiSeq Illumina real-time analysis (RTA) software v1.18.61 update. This software generates a color matrix for the correction of the reads. This is important because HiSeq sequencer uses different lasers to detect G/T and A/C nucleotides. In each cycle, at least one of two nucleotides for each color channel must be read in order to maintain the color balance for each base in the index read sequenced. With this upgrade the color matrix still uses the first four cycles to generate data, like the last version of RTA. However, in the current version the initial matrix is discarded after the template generation is complete. Then, the first 11 cycles of intensity data are used for final estimation of the correction matrix. In order to minimize the issues related to the construction of this matrix, we optimized our protocol using barcodes larger than 4 bp to avoid imbalance between the first bases. The complete laboratory procedures are provided in Supplementary Data S1.

### Sequence processing

Quality trimming was performed in short sequences with SeqyClean tool v. 1.9.10^18^ using a Phred quality score ≥24 and a fragment size ≥50. The quality of the *reads* was checked before and after the cleaning by FastQC v.0.11.3^38^.

The Tassel v.3.0 program was used to process the data^7^. For each sample stored in a FASTQ file there is one identification map key file. This key file has the matching information for the sample, flowcell and lane. The reads that begin with one of the expected barcodes (found in the key map) are immediately followed by the expected cut site remnant (CTGCA for *PstI*). Fragments are then trimmed to 64 bases and grouped into a single list called “master” by the TASSEL-GBS Discovery Pipeline.

### Alignment and Genetic variants identification

The alignment of quality-trimmed reads was performed using Bowtie2 tool v.2.2.5^39^ against the current chicken reference sequence (*Gallus_gallus* 4.0, NCBI). The aligned reads were then imputed in the Tassel v.3.0 default pipeline^7^ for SNP identification. We filtered the polymorphisms initially identified based on the sequencing quality criteria and on the bases identified. The following filters were applied: i) minimum taxon call rate (mnTCov) of 20%, which is a minimum SNP call rate for a taxon to be included in the output, with the call rate being the proportion of the SNP genotypes for a non-N taxon (where N = missing); ii) minimum site coverage (mnScov) of 90%, which is a minimum taxon call rate for a SNP to be included in the output, with the taxon call rate being the proportion of the taxa with non-N genotypes for that SNP; iii) mismatch rate (misMat) of 5% to minimize the appearance of duplicated SNPs; iv) minimum minor allele frequency (mnMAF) of 0.01. A more detailed description of the default filters has been provided by Glaubitz *et al.* (2014).

The coverage depth of the “unique sequence tags” file was determined using Samtools v.0.1.19^40^ with the “depth” option.

### Genotyping methods comparison and CornellGBS data validation

We compared the chromosomal positions of the SNPs obtained using the CornellGBS approach with the positions obtained using the following SNP platforms for chickens: Illumina Chicken 60K Beadchip^25^ and 600K HD Affymetrix^®^Axion^®^ genotyping array for chicken^3^. Bioconductor^37^ (GEOquery) and CRAN (data.table, rdrop2 and reshape) repository packages for R were used for the bioinformatics analysis. We validated our method comparing the SNPs obtained (59,205) against a SNP dataset of WCGR (Boschiero *et al.*, unpublished results) previously generated with Illumina sequencing with ~11X of sequencing coverage. This dataset contained 12,357,602 filtered SNPs and was generated from the same 10 chickens used in this study (TT and CC parental lines). The comparison between these two datasets was performed using CRAN (data.table and reshape2) repository packages for R. More details of the sequencing process of WCGR SNP data can be found in recent publications^31^,^32^.

### Functional annotation

The set of unique SNPs obtained from 462 chickens using the Tassel v.3.0 tool was annotated using the Variant Effect Predictor (VEP) tool v.71^41^. The SIFT (sorting intolerant from tolerant) scores for the SNPs^33^ were used to predict whether a substitution of an amino acid affects protein function, which is based on sequence homology and the physical properties of amino acids. If the SIFT score lies at or below the 0.05 threshold, the variant causing the amino acid was considered non tolerated.

### Mendelian inheritance of the SNPs

The Mendelian error testing was performed using SNP & Variation Suite v8.4^42^.

### Genetic map construction

SNPs present in all families were filtering using Tassel program^7^. A pseudo-testcross population was used to construct the F_1_ linkage map. For the linkage analysis, the SNPs were first tested against the expected segregation ratio. The informative genotypes combination were selected for the map construction. Markers with significant segregation distortion (P < 0.001, χ2 test) were removed.

The genetic map was constructed using R/OneMap package^43^ and JoinMap v.4.1^44^. The R/OneMap was used to join the markers in the linkage groups (LGs). The minimum LOD values of 8 and a maximum recombination fraction of 0.35 were used to organize the markers in each LG with the regression mapping algorithm and the Kosambi mapping function^45^.

The R/OmicCircus package^46^ was used to plot the relationship between the chromosomal and linkage marker groups formed by the abovementioned genetic map.

## Additional Information

**How to cite this article**: Pértille, F. *et al.* High-throughput and Cost-effective Chicken Genotyping Using Next-Generation Sequencing. *Sci. Rep.* **6**, 26929; doi: 10.1038/srep26929 (2016).

## Supplementary Material

Supplementary Information

Supplementary Spreadsheet S1

## Figures and Tables

**Table 1:** Basic statistical parameters of SNPs distance.

Platform	Mean (Kb)	Median (Kb)	Min (bp)	Max (Mb)
600K Affymetrix	1.7	1.2	1	1.4
CornellGBS	15	0.05	1	1.8
60K Illumina	21	18	1	2

SNPs were mapped against chromosome locations of the chicken genome after being detected with 600K Affymetrix, CornellGBS or 60K Illumina.

**Table 2:** Proportion of SNPs detected in each chromosomal size category after using three different genotyping platforms: 600K Affymetrix, CornellGBS and 60K Illumina.

Platform	Large GGA%	Medium GGA%	Micro GGA %
600K Affymetrix	54.81	17.00	28.19
CornellGBS	33.50	16.37	50.13
60K Illumina	50.30	17.00	32.70

**Table 3:** SNP heterozygosity of the genotyped populations (parental, F_1_ and F_2_ generations).

Population	Number of individuals	Number Heterozygous SNPs	Proportion heterozygous (SD)		CV
Paternal CC	5	11888	0.20	(±0.01)	3%
Paternal TT	5	15244	0.26	(±0.01)	4%
F_1_	8	20101	0.32	(±0.10)	30%
F_2_-7765	72	21658	0.33	(±0.03)	8%
F_2_-7810	82	20323	0.31	(±0.03)	11%
F_2_-7816	94	22016	0.34	(±0.09)	25%
F_2_-7971	100	18865	0.29	(±0.05)	16%
F_2_-7978	96	19982	0.30	(±0.03)	11%

**Table 4:** Annotation results of the complete set of 67,096 PstI-derived SNPs (after filtering) obtained after genotyping 462 chickens.

Variants	Total no.	%
*All variants*	78,399	100
Intronic	28,181	35.95
Intergenic	22,116	28.21
Exonic	2,590	3.30
Splicing	256	0.11
ncRNA	6	0.01
5′-UTR	268	0.34
3′-UTR	1,328	1.69
Upstream (1kb)	11,516	14.69
Downstream (1kb)	12,306	15.70
*Exonic*		
Synonymous	1,671	64.52
Non-synonymous	907	35.02
Startlost	5	0.19
Stopgain	3	0.12
Stoplost	4	0.15

**Table 5:** Assessment of Mendelian errors in the dataset of 67,096 *PstI*-derived SNPs (after filtering) and in the subset of 13,434 novel SNPs identified.

Family	N° of Individuals	Mendelian Errors	% of markers	Mendelian Errors (novelSNPs)	% of markers
**F1**	8	6,488 ± 2,835	9.7	1,313 ± 567	9.7
**F2**	444	5,947 ± 1,169	8.9	1,216 ± 609	9.0
F2-7765	72	5,060 ± 1,527	7.5	1,039 ± 633	7.7
F2-7810	82	5,464 ± 1,084	8.1	1,124 ± 695	8.3
F2-7816	94	7,872 ± 919	11.7	1,575 ± 757	11.6
F2-7971	100	5,125 ± 471	7.6	1,075 ± 907	7.9
F2-7978	96	6,212 ± 918	9.3	1,268 ± 71	9.4
**Total Analysed**	452	67,096		13,543	

Results are shown separately for each generation of animals studied and for the different families within the F_2_ generation population.
