# Supplementary material for: High-throughput and Cost-effective Chicken Genotyping Using Next-Generation Sequencing
Source: Sci Rep. 2016 May 25;6:26929. doi: 10.1038/srep26929 (PMC4879531; doi:10.1038/srep26929)
Supplement: Supplementary Information [file srep26929-s1.doc]

High-throughput and Cost-effective Chicken Genotyping Using Next-Generation Sequencing

Fábio Pértille^1*^; Carlos Guerrero-Bosagna^2^; Vinicius Henrique da Silva^1^; Clarissa Boschiero^1^; José Ribamar Nunes^1^; Mônica Corrêa Ledur^3^; Per Jensen^2^ & Luiz Lehmann Coutinho^1^

^1^ Animal Biotechnology Laboratory, Animal Science and Pastures Department, University of São Paulo (USP)/ Luiz de Queiroz College of Agriculture (ESALQ), Piracicaba, São Paulo, Brazil

^2^ IFM Biology, AVIAN Behavioural Genomics and Physiology Group, Linköping University, Linköping, Sweden

^3^ Brazilian Agricultural Research Corporation (EMBRAPA) Swine & Poultry, Concórdia, Santa Catarina, Brazil

*Corresponding author:

Email: [pertille@usp.br](mailto:pertille@usp.br) (FP)

**SUPPLEMENTARY INFORMATION**

**1. Supplementary figures**

Supplementary Fig. S1. Representation of the Karyotype distribution of specific cut-site fragments (ranging 300 and 500 bp) generated by *PstI* or *SbfI* restriction enzymes throughout the chicken genome. *PstI* restriction enzyme cleavage (red) generates 100,199 fragments, while *SbfI* restriction enzyme cleavage (blue) generates 809 fragments.

Supplementary Fig. S2. Agarose gel electrophoresis (1%) showing in the first two lanes the cleavage of a chicken DNA sample using *PstI* or *SbfI* restriction enzymes. The Φ*X174 DNA-Hae II Digest* ladder (New England BioLabs™) is shown in the third lane.

Supplementary Fig. S3. Agarose gel electrophoresis (1%) showing the result of amplified DNA pools (a) before and (b) after the DNA purification by the Agencourt®AMPure®XP protocol . Low DNA Mass Ladder (Invitrogen™) is included in the third lane in (b).

Supplementary Fig. S4. Distribution of the number of sequenced reads in individual DNA samples sequenced in each flow cell lane.

Supplementary Fig. S5. Framework consensus chicken linkage map obtained from 4,469 *PstI*-derived SNPs organized in 29 linkage groups built from 5 common markers in the F_2_ families. The length in cM is displayed to the left. Names and distance between markers are displayed at scale within each group (provided separately in pdf format).

**2. Supplementary tables**

Supplementary Table S1. Number of *Predicted* *PstI-Tags* and *Predicted* *SbfI-Tags* obtained from the *in silico* chicken genome cleavage using *PstI* or *SbfI* ; also number of Sequenced *PstI-Tags*  generated from the alignment of 462 chickens against the chicken genome and corresponding percentages.

Supplementary Table S2. Number and proportion of alleles obtained by the SNP calls

Supplementary Table S3. Description of 155 non-tolerated SNPs identified in chickens

Supplementary Table S4. Number of genes, SNPs and SNP densities per chromosome, obtained after genotyping with the CornellGBS, 60K Illumina or 600K Affymetrix platforms.

Supplementary Table S5. Annotation of unique 67,096 PstI-derived SNPs from 462 chickens (CornellGBS), unique 51,343 SNPs obtained with 60 K Illumina and unique 618,308 SNPs obtained with 600K Affymetrix.

**3. Supplementary data**

Supplementary Data S1. CornellGBS workflow

**4. Supplementary spreadsheet**

Supplementary Spreadsheet S1. Functional annotation of novel SNPs detected by CornellGBS (provided separately in .xls format)

**1. Supplementary figures**

Supplementary Fig. S1.


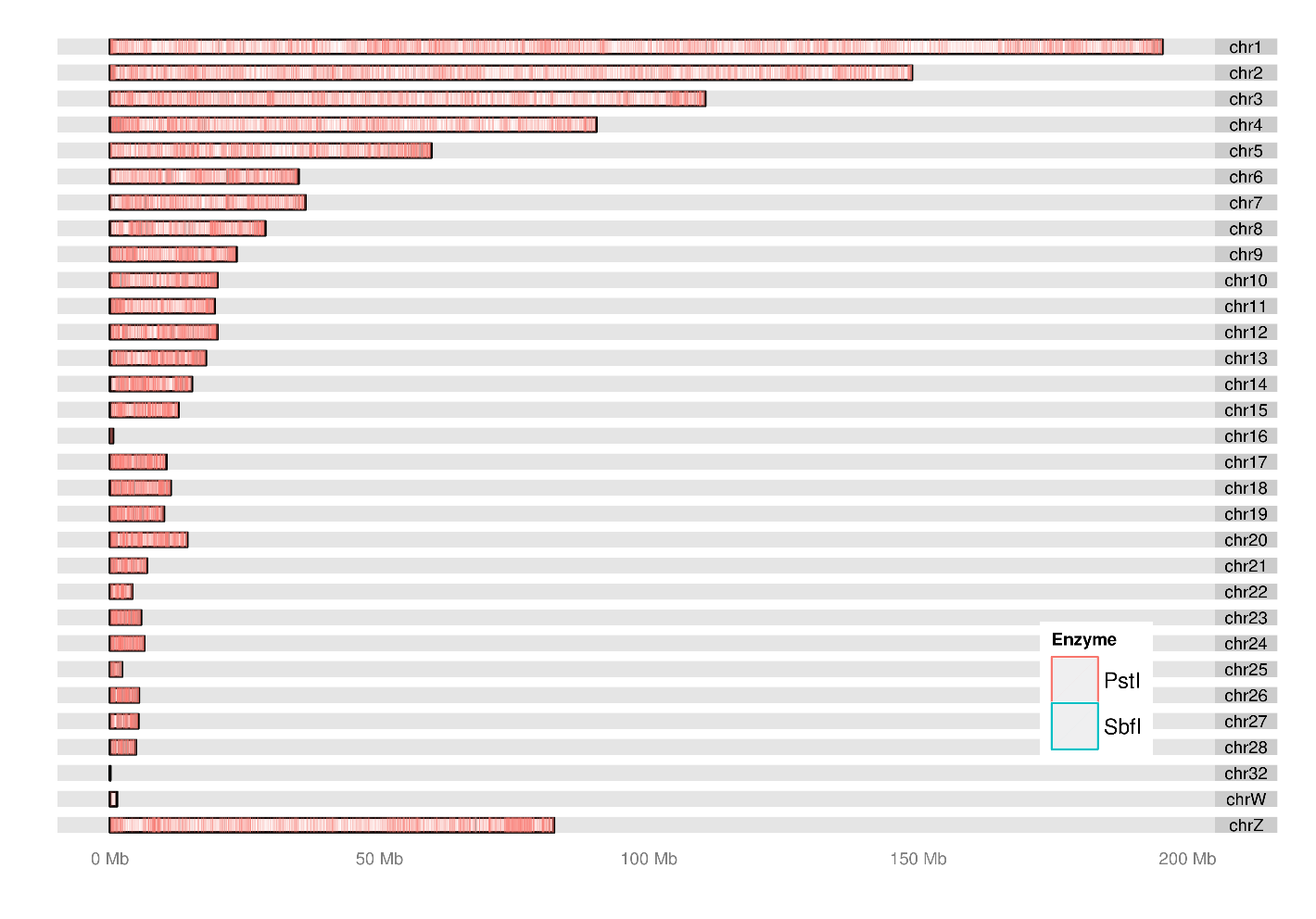


Supplementary Fig. S2.


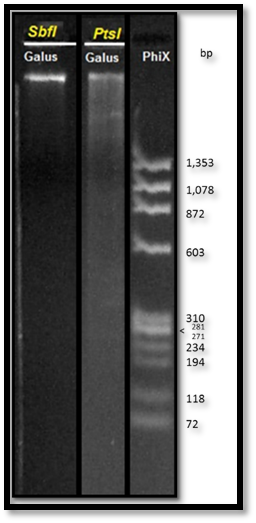


Supplementary Fig. S3.


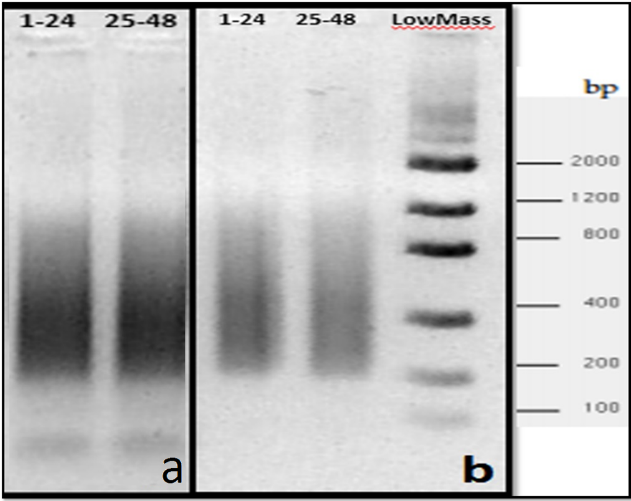


Supplementary Fig. S4.
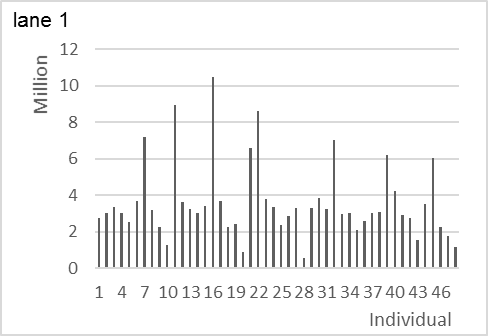

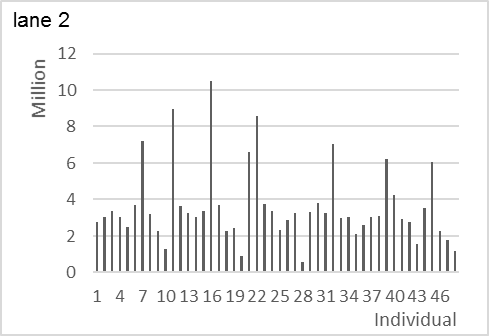

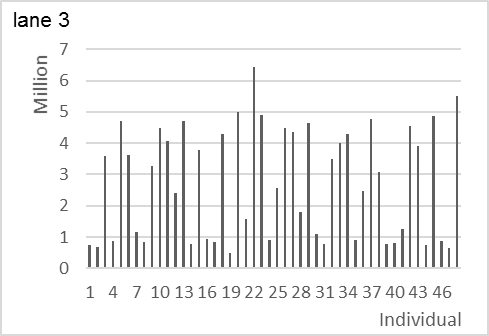

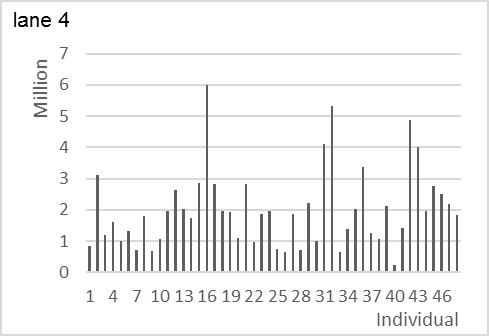

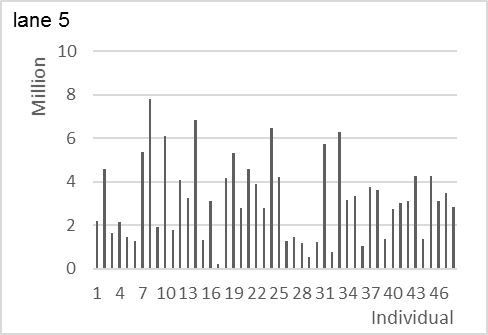

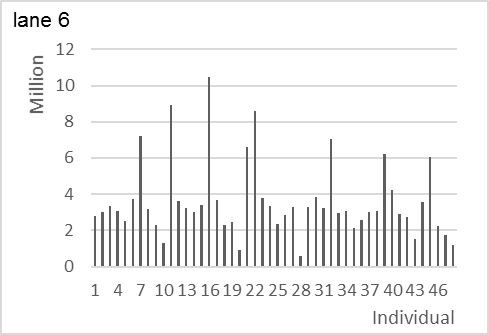

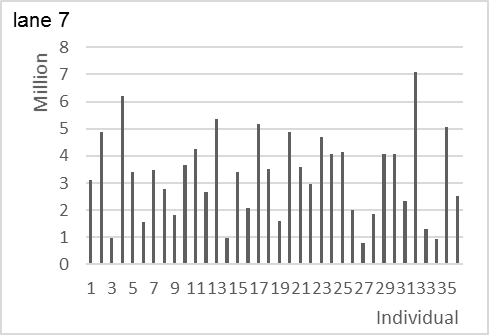

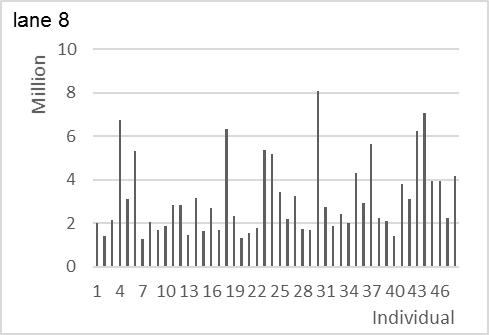

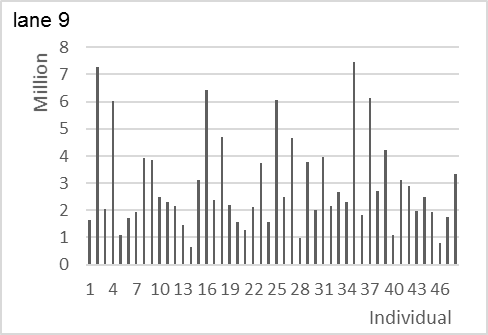

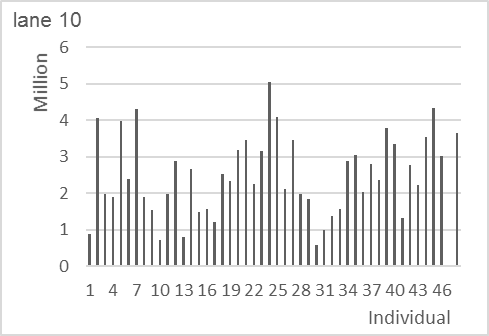


**2. Supplementary tables**

Supplementary Table S1.

|  | ***PstI* predicted** | **%** | ***PstI* sequenced** | **%** | ***SbfI* predicted** | **%** |
| --- | --- | --- | --- | --- | --- | --- |
| **All** | 811951 | 100 | 287819 | 100 | 45116 | 100 |
| **0-15000** | 811625 | 99.96 | 291498 | 101.28 | 26922 | 59.67 |
| **0-100** | 93409 | 11.50 | 34808 | 12.09 | 498 | 1.10 |
| **101-200** | 70394 | 8.67 | 30168 | 10.48 | 412 | 0.91 |
| **201-300** | 61003 | 7.51 | 26515 | 9.21 | 385 | 0.85 |
| **301-400** | 52881 | 6.51 | 23257 | 8.08 | 396 | 0.88 |
| **401-500** | 45789 | 5.64 | 20617 | 7.16 | 405 | 0.90 |
| **501-600** | 40950 | 5.04 | 18792 | 6.53 | 372 | 0.82 |
| **601-700** | 36436 | 4.49 | 15804 | 5.49 | 357 | 0.79 |
| **701-800** | 32600 | 4.02 | 11494 | 3.99 | 353 | 0.78 |
| **801-900** | 29370 | 3.62 | 8162 | 2.84 | 328 | 0.73 |
| **901-1000** | 26768 | 3.30 | 6950 | 2.41 | 340 | 0.75 |
| **1001-2000** | 167655 | 20.65 | 45572 | 15.83 | 3430 | 7.60 |
| **2001-3000** | 74549 | 9.18 | 21528 | 7.48 | 2786 | 6.18 |
| **3001-4000** | 36631 | 4.51 | 10707 | 3.72 | 2267 | 5.02 |
| **4001-5000** | 18908 | 2.33 | 5732 | 1.99 | 2138 | 4.74 |
| **5001-6000** | 10322 | 1.27 | 3244 | 1.13 | 1875 | 4.16 |
| **6001-7000** | 5785 | 0.71 | 1792 | 0.62 | 1628 | 3.61 |
| **7001-8000** | 3379 | 0.42 | 1037 | 0.36 | 1513 | 3.35 |
| **8001-9000** | 1904 | 0.23 | 610 | 0.21 | 1314 | 2.91 |
| **9001-10000** | 1168 | 0.14 | 380 | 0.13 | 1255 | 2.78 |
| **10001-11000** | 696 | 0.09 | 224 | 0.08 | 1106 | 2.45 |
| **11001-12000** | 443 | 0.05 | 142 | 0.05 | 1045 | 2.32 |
| **12001-13000** | 286 | 0.04 | 84 | 0.03 | 997 | 2.21 |
| **13001-14000** | 174 | 0.02 | 52 | 0.02 | 888 | 1.97 |
| **14001-15000** | 125 | 0.02 | 36 | 0.01 | 834 | 1.85 |
| **>15001** | 326 | 0.04 | 112 | 0.04 | 18194 | 40.33 |

Supplementary Table S1.

| **Alleles** | **Number** | **Proportion** | **Alleles** | **Number** | **Proportion** |
| --- | --- | --- | --- | --- | --- |
| G | 6,365,808 | 0.21 | G:A | 14,938 | 0.22 |
| C | 6,331,609 | 0.20 | C:T | 14,733 | 0.22 |
| T | 4,001,626 | 0.13 | T:C | 9,080 | 0.14 |
| A | 3,930,142 | 0.13 | A:G | 8,895 | 0.13 |
| R | 3,342,860 | 0.11 | C:G | 3,110 | 0.05 |
| Y | 3,342,552 | 0.11 | G:C | 3,045 | 0.05 |
| N | 972,592 | 0.03 | G:T | 2,835 | 0.04 |
| S | 872,322 | 0.03 | C:A | 2,820 | 0.04 |
| K | 679,087 | 0.02 | T:G | 2,086 | 0.03 |
| M | 666,021 | 0.02 | A:C | 2,059 | 0.03 |
| W | 493,733 | 0.02 | T:A | 1,783 | 0.03 |
| **Tota**l | 30,998,352 |  | A:T | 1,712 | 0.03 |
|  | (taxa*site) | 100% |  | 65,384(sites) | 97.4% |

Supplementary Table S2.

| **Chr** | **Postion (pb)** | **SNP** | **Amino acids changed** | **SIFT score** | **Existing variation (dbSNP ID)** | **Gene Symbol** | **cDNA position** |
| --- | --- | --- | --- | --- | --- | --- | --- |
| 1 | 405533 | C/T | A/V | 0.01 | - | [novel gene](http://www.ensembl.org/Gallus_gallus/Gene/Summary?g=ENSGALG00000026110&db=core) | 2390 |
| 1 | 412000 | A/G | S/G | 0.04 | - | [novel gene](http://www.ensembl.org/Gallus_gallus/Gene/Summary?g=ENSGALG00000026110&db=core) | 3472 |
| 1 | 412036 | C/G | P/A | 0 | - | [novel gene](http://www.ensembl.org/Gallus_gallus/Gene/Summary?g=ENSGALG00000026110&db=core) | 3508 |
| 1 | 1029019 | C/T | A/T | 0.01 | - | *FAM208B* | 8064 |
| 1 | 49286694 | C/T | R/C | 0 | rs316798537 | *NAGA* | 881 |
| 1 | 60067442 | A/G | T/A | 0.05 | rs13874831 | *DDX11* | 1019 |
| 1 | 67780579 | C/A | S/Y | 0.01 | rs313846534 | SSPN | 588 |
| 1 | 76711976 | C/T | R/C | 0 | - | GPR162 | 2470 |
| 1 | 91236887 | C/T | R/C | 0 | - | ILDR2 | 1063 |
| 1 | 91438194 | A/T | K/M | 0.01 | rs313179097 | CD101 | 2771 |
| 1 | 110700007 | T/C | V/A | 0.01 | - | [novel gene](http://www.ensembl.org/Gallus_gallus/Gene/Summary?g=ENSGALG00000022787&db=core) | 1793 |
| 1 | 113135717 | A/C | V/G | 0.02 | - | MROH8 | 2012 |
| 1 | 113135730 | C/T | G/R | 0 | - | MROH8 | 1999 |
| 1 | 116327699 | C/T | G/R | 0 | - | [novel gene](http://www.ensembl.org/Gallus_gallus/Gene/Summary?g=ENSGALG00000016290&db=core) | 1522 |
| 1 | 116327700 | C/G | R/S | 0 | - | [novel gene](http://www.ensembl.org/Gallus_gallus/Gene/Summary?g=ENSGALG00000016290&db=core) | 1521 |
| 1 | 116327702 | T/C | R/G | 0 | - | [novel gene](http://www.ensembl.org/Gallus_gallus/Gene/Summary?g=ENSGALG00000016290&db=core) | 1519 |
| 1 | 116327706 | C/A | Q/H | 0.01 | - | [novel gene](http://www.ensembl.org/Gallus_gallus/Gene/Summary?g=ENSGALG00000016290&db=core) | 1515 |
| 1 | 116327707 | T/C | Q/R | 0.05 | - | [novel gene](http://www.ensembl.org/Gallus_gallus/Gene/Summary?g=ENSGALG00000016290&db=core) | 1514 |
| 1 | 116327708 | G/C | Q/E | 0.02 | - | [novel gene](http://www.ensembl.org/Gallus_gallus/Gene/Summary?g=ENSGALG00000016290&db=core) | 1513 |
| 1 | 116327722 | C/A | R/M | 0.01 | - | [novel gene](http://www.ensembl.org/Gallus_gallus/Gene/Summary?g=ENSGALG00000016290&db=core) | 1499 |
| 1 | 116327723 | T/C | R/G | 0.02 | - | [novel gene](http://www.ensembl.org/Gallus_gallus/Gene/Summary?g=ENSGALG00000016290&db=core) | 1498 |
| 1 | 116327734 | A/C | L/R | 0 | - | [novel gene](http://www.ensembl.org/Gallus_gallus/Gene/Summary?g=ENSGALG00000016290&db=core) | 1487 |
| 1 | 116327735 | G/T | L/M | 0.02 | - | [novel gene](http://www.ensembl.org/Gallus_gallus/Gene/Summary?g=ENSGALG00000016290&db=core) | 1486 |
| 1 | 116327738 | T/C | K/E | 0.05 | - | [novel gene](http://www.ensembl.org/Gallus_gallus/Gene/Summary?g=ENSGALG00000016290&db=core) | 1483 |
| 1 | 121027885 | C/G | G/R | 0 | - | [novel gene](http://www.ensembl.org/Gallus_gallus/Gene/Summary?g=ENSGALG00000026840&db=core) | 1705 |
| 1 | 121027923 | T/G | K/T | 0.03 | - | [novel gene](http://www.ensembl.org/Gallus_gallus/Gene/Summary?g=ENSGALG00000026840&db=core) | 1667 |
| 1 | 129829303 | G/T | A/D | 0 | - | NIPA1 | 188 |
| 1 | 139404558 | G/C | E/Q | 0 | - | [novel gene](http://www.ensembl.org/Gallus_gallus/Gene/Summary?g=ENSGALG00000013985&db=core) | 4144 |
| 1 | 139404574 | G/A | R/Q | 0 | - | [novel gene](http://www.ensembl.org/Gallus_gallus/Gene/Summary?g=ENSGALG00000013985&db=core) | 4160 |
| 1 | 139404579 | A/G | K/E | 0 | - | [novel gene](http://www.ensembl.org/Gallus_gallus/Gene/Summary?g=ENSGALG00000013985&db=core) | 4165 |
| 1 | 139404889 | C/T | T/I | 0 | - | [novel gene](http://www.ensembl.org/Gallus_gallus/Gene/Summary?g=ENSGALG00000013985&db=core) | 4475 |
| 1 | 193363849 | G/C | S/T | 0.03 | - | [novel gene](http://www.ensembl.org/Gallus_gallus/Gene/Summary?g=ENSGALG00000017493&db=core) | 648 |
| 1 | 193363851 | C/A | P/T | 0.02 | - | [novel gene](http://www.ensembl.org/Gallus_gallus/Gene/Summary?g=ENSGALG00000017493&db=core) | 650 |
| 1 | 193363863 | A/C | S/R | 0 | - | [novel gene](http://www.ensembl.org/Gallus_gallus/Gene/Summary?g=ENSGALG00000017493&db=core) | 662 |
| 1 | 193363870 | C/T | P/L | 0.02 | - | [novel gene](http://www.ensembl.org/Gallus_gallus/Gene/Summary?g=ENSGALG00000017493&db=core) | 669 |
| 1 | 193367392 | G/A | S/L | 0.05 | - | NUMA1 | 5208 |
| 1 | 194503705 | G/A | R/H | 0.01 | - | ARHGEF17 | 233 |
| 2 | 564412 | G/T | A/S | 0.05 | - | [novel gene](http://www.ensembl.org/Gallus_gallus/Gene/Summary?g=ENSGALG00000004931&db=core) | 383 |
| 2 | 76305835 | C/T | A/V | 0.02 | - | [novel gene](http://www.ensembl.org/Gallus_gallus/Gene/Summary?g=ENSGALG00000026079&db=core) | 113 |
| 2 | 81744245 | T/A | K/N | 0 | - | [novel gene](http://www.ensembl.org/Gallus_gallus/Gene/Summary?g=ENSGALG00000026637&db=core) | 812 |
| 3 | 74949369 | G/A | V/I | 0.05 | rs10725232 | LYRM2 | 121 |
| 3 | 107247434 | C/T | S/F | 0 | rs313012221 | PKHD1 | 1031 |
| 4 | 3435927 | T/G | R/S | 0 | rs316726784 | FRMD7 | 1941 |
| 4 | 8073777 | C/T | E/K | 0.04 | - | KLHL4 | 527 |
| 4 | 10974825 | G/A | P/L | 0.01 | - | [novel gene](http://www.ensembl.org/Gallus_gallus/Gene/Summary?g=ENSGALG00000023692&db=core) | 471 |
| 4 | 49130705 | C/G | A/G | 0 | - | SOWAHB | 1088 |
| 4 | 49130707 | A/C | K/Q | 0 | - | SOWAHB | 1090 |
| 4 | 85381698 | T/C | T/A | 0.02 | - | [novel gene](http://www.ensembl.org/Gallus_gallus/Gene/Summary?g=ENSGALG00000022939&db=core) | 484 |
| 4 | 89105757 | G/A | R/W | 0.01 | - | ADAM33 | 256 |
| 5 | 225973 | C/G | H/Q | 0 | - | [novel gene](http://www.ensembl.org/Gallus_gallus/Gene/Summary?g=ENSGALG00000003608&db=core) | 723 |
| 5 | 15151421 | C/T | R/W | 0 | rs16752593 | PIDD1 | 795 |
| 5 | 15481464 | G/T | M/I | 0.04 | rs315606489 | RNH1 | 52 |
| 5 | 16172098 | T/C | F/S | 0 | - | [novel gene](http://www.ensembl.org/Gallus_gallus/Gene/Summary?g=ENSGALG00000010831&db=core) | 2162 |
| 5 | 16172101 | G/T | G/V | 0.04 | - | [novel gene](http://www.ensembl.org/Gallus_gallus/Gene/Summary?g=ENSGALG00000010831&db=core) | 2165 |
| 5 | 16172104 | T/C | V/A | 0.04 | - | [novel gene](http://www.ensembl.org/Gallus_gallus/Gene/Summary?g=ENSGALG00000010831&db=core) | 2168 |
| 5 | 16172485 | C/G | L/V | 0.03 | - | [novel gene](http://www.ensembl.org/Gallus_gallus/Gene/Summary?g=ENSGALG00000010831&db=core) | 2452 |
| 5 | 16172510 | C/T | T/I | 0.01 | - | [novel gene](http://www.ensembl.org/Gallus_gallus/Gene/Summary?g=ENSGALG00000010831&db=core) | 2477 |
| 5 | 16203346 | A/C | T/P | 0.02 | - | [novel gene](http://www.ensembl.org/Gallus_gallus/Gene/Summary?g=ENSGALG00000028071&db=core) | 547 |
| 5 | 57816830 | A/G | L/P | 0.01 | - | NIN | 4466 |
| 6 | 5484797 | C/T | A/T | 0 | - | [novel gene](http://www.ensembl.org/Gallus_gallus/Gene/Summary?g=ENSGALG00000024315&db=core) | 271 |
| 6 | 8488012 | G/T | A/S | 0 | rs317408356 | chAnk3 | 5266 |
| 6 | 11068571 | C/T | A/V | 0.02 | - | SPOCK2 | 425 |
| 6 | 11135860 | G/A | R/C | 0.02 | rs312551127 | [novel gene](http://www.ensembl.org/Gallus_gallus/Gene/Summary?g=ENSGALG00000004497&db=core) | 487 |
| 6 | 21862128 | G/A | R/C | 0.01 | - | HOGA1 | 793 |
| 6 | 25343255 | A/T | K/M | 0 | - | ADD3 | 1093 |
| 6 | 30932171 | G/T | A/D | 0 | - | C10orf88 | 317 |
| 7 | 5139857 | C/G | E/Q | 0.02 | rs314384300 | AGAP1 | 1825 |
| 7 | 15764099 | G/T | L/M | 0.01 | - | HOXD3 | 1069 |
| 8 | 24183743 | G/A | C/Y | 0.01 | rs318108723 | PCSK9 | 1693 |
| 9 | 4692523 | A/G | T/A | 0.05 | - | TSPEAR | 682 |
| 9 | 12393793 | C/T | R/C | 0 | - | CPN2 | 1325 |
| 10 | 267173 | G/T | V/F | 0.02 | - | [novel gene](http://www.ensembl.org/Gallus_gallus/Gene/Summary?g=ENSGALG00000029029&db=core) | 646 |
| 10 | 267218 | G/C | V/L | 0 | - | [novel gene](http://www.ensembl.org/Gallus_gallus/Gene/Summary?g=ENSGALG00000029029&db=core) | 691 |
| 10 | 1841408 | G/A | S/L | 0.01 | rs317653103 | [novel gene](http://www.ensembl.org/Gallus_gallus/Gene/Summary?g=ENSGALG00000014107&db=core) | 617 |
| 10 | 2084689 | C/T | E/K | 0 | - | CCDC33 | 61 |
| 10 | 2116734 | C/T | P/L | 0.03 | - | STOML1 | 889 |
| 10 | 4904503 | C/A | R/L | 0.01 | - | GCNT3 | 707 |
| 10 | 10161148 | G/C | R/P | 0.05 | rs15576100 | SPATA5L1 | 383 |
| 10 | 11576105 | T/C | L/P | 0.02 | rs314957346 | TMC3 | 398 |
| 10 | 19330662 | G/A | S/F | 0 | - | CASC4 | 323 |
| 10 | 19533141 | G/A | R/Q | 0.05 | - | SEMA4B | 960 |
| 10 | 19533165 | A/G | Q/R | 0 | - | SEMA4B | 984 |
| 10 | 19533191 | G/C | G/R | 0.04 | - | SEMA4B | 1010 |
| 10 | 19533192 | G/A | G/D | 0 | - | SEMA4B | 1011 |
| 10 | 19778176 | G/A | D/N | 0 | - | TP53BP1 | 5923 |
| 10 | 19778188 | G/A | A/T | 0.03 | - | TP53BP1 | 5935 |
| 11 | 1249258 | C/T | S/N | 0 | - | PLEKHG4 | 4115 |
| 11 | 2449318 | T/C | L/P | 0 | - | FBXL8 | 311 |
| 11 | 2649515 | C/A | H/Q | 0.04 | - | [novel gene](http://www.ensembl.org/Gallus_gallus/Gene/Summary?g=ENSGALG00000021324&db=core) | 63 |
| 11 | 2649563 | G/A | M/I | 0.04 | - | [novel gene](http://www.ensembl.org/Gallus_gallus/Gene/Summary?g=ENSGALG00000021324&db=core) | 111 |
| 11 | 2649604 | T/C | I/T | 0.02 | - | [novel gene](http://www.ensembl.org/Gallus_gallus/Gene/Summary?g=ENSGALG00000021324&db=core) | 152 |
| 11 | 2649609 | C/A | L/M | 0.01 | - | [novel gene](http://www.ensembl.org/Gallus_gallus/Gene/Summary?g=ENSGALG00000021324&db=core) | 157 |
| 11 | 2649615 | G/T | A/S | 0.01 | - | [novel gene](http://www.ensembl.org/Gallus_gallus/Gene/Summary?g=ENSGALG00000021324&db=core) | 163 |
| 11 | 2649618 | C/T | L/F | 0.01 | - | [novel gene](http://www.ensembl.org/Gallus_gallus/Gene/Summary?g=ENSGALG00000021324&db=core) | 166 |
| 11 | 18288252 | G/A | V/I | 0.04 | rs316651020 | MC1R | 376 |
| 11 | 18288274 | T/A | L/Q | 0 | - | MC1R | 398 |
| 11 | 18291169 | T/G | C/G | 0 | - | TUBB3 | 211 |
| 11 | 18291171 | T/G | C/W | 0 | - | TUBB3 | 213 |
| 12 | 2423621 | G/A | A/V | 0 | rs314122666 | CDHR4 | 737 |
| 12 | 2489639 | G/C | S/C | 0.03 | - | APEH | 1148 |
| 12 | 11558612 | C/T | R/W | 0.02 | - | USP19 | 127 |
| 13 | 373892 | A/G | I/V | 0.05 | - | HARS | 803 |
| 13 | 972934 | C/G | P/A | 0 | rs14049768 | SLC4A9 | 163 |
| 13 | 1807505 | G/A | S/N | 0.04 | - | MZB1 | 383 |
| 14 | 13260080 | T/C | Q/R | 0.05 | - | PPL | 2600 |
| 15 | 1300093 | A/T | L/Q | 0 | - | TRMT2A | 444 |
| 15 | 9516709 | G/A | T/M | 0.01 | rs317964243 | CCDC64 | 680 |
| 15 | 9992022 | C/G | D/H | 0.03 | - | SELM | 319 |
| 15 | 10853170 | G/A | G/D | 0.01 | rs15025563 | GATSL3 | 275 |
| 16 | 162855 | C/A | C/F | 0.05 | - | TRIM27.2 | 128 |
| 16 | 226018 | G/A | S/L | 0 | rs314003274 | KIFC1 | 1255 |
| 16 | 227749 | T/G | T/P | 0.01 | - | KIFC1 | 723 |
| 16 | 227764 | T/C | R/G | 0.01 | - | KIFC1 | 708 |
| 16 | 227772 | T/C | E/G | 0.05 | - | KIFC1 | 700 |
| 17 | 559222 | C/T | A/T | 0 | rs312924788 | MAMDC4 | 1915 |
| 17 | 1050470 | C/T | T/M | 0.03 | rs317932652 | [novel gene](http://www.ensembl.org/Gallus_gallus/Gene/Summary?g=ENSGALG00000008891&db=core) | 1211 |
| 17 | 7841652 | C/G | P/R | 0.01 | - | NOTCH1 | 7094 |
| 18 | 2183196 | G/A | R/Q | 0.04 | - | USP43 | 927 |
| 18 | 4687954 | C/G | M/I | 0.02 | - | [novel gene](http://www.ensembl.org/Gallus_gallus/Gene/Summary?g=ENSGALG00000002290&db=core) | 435 |
| 19 | 3415614 | C/A | K/N | 0.02 | - | MIS12 | 564 |
| 19 | 3415832 | G/A | R/C | 0.05 | rs431855703 | MIS12 | 346 |
| 19 | 4506372 | C/T | R/H | 0 | rs431884891 | LIG3 | 2474 |
| 19 | 5741294 | C/G | V/L | 0.01 | - | [novel gene](http://www.ensembl.org/Gallus_gallus/Gene/Summary?g=ENSGALG00000026433&db=core) | 2512 |
| 19 | 5880142 | T/C | T/A | 0.01 | rs315563948 | DHRS13 | 838 |
| 19 | 5944415 | C/A | S/I | 0.01 | - | MYO18A | 2417 |
| 19 | 7197940 | C/T | P/L | 0.01 | - | PRR11 | 412 |
| 20 | 8299946 | C/T | G/R | 0.01 | - | CABLES2 | 442 |
| 20 | 8299954 | G/T | A/D | 0.01 | - | CABLES2 | 434 |
| 20 | 10667933 | A/G | S/G | 0.01 | rs316979376 | BPIFB4 | 1234 |
| 20 | 10701591 | A/T | Q/L | 0.04 | - | KIAA1755 | 941 |
| 20 | 12430772 | C/G | P/R | 0.03 | - | [novel gene](http://www.ensembl.org/Gallus_gallus/Gene/Summary?g=ENSGALG00000020895&db=core) | 1132 |
| 21 | 4875212 | G/T | S/I | 0 | - | [novel gene](http://www.ensembl.org/Gallus_gallus/Gene/Summary?g=ENSGALG00000010242&db=core) | 1351 |
| 22 | 2234762 | T/C | D/G | 0.01 | rs317879584 | [novel gene](http://www.ensembl.org/Gallus_gallus/Gene/Summary?g=ENSGALG00000021824&db=core) | 359 |
| 23 | 4094862 | G/A | R/C | 0 | - | ADPRHL2 | 877 |
| 23 | 4831549 | G/A | P/L | 0 | - | COL9A2 | 564 |
| 23 | 5413219 | G/A | D/N | 0.02 | rs15207937 | [novel gene](http://www.ensembl.org/Gallus_gallus/Gene/Summary?g=ENSGALG00000024064&db=core) | 776 |
| 25 | 1369672 | G/A | R/H | 0.04 | - | FDPS | 521 |
| 25 | 1588159 | C/T | V/M | 0.01 | - | FCRL2 | 1249 |
| 26 | 464991 | G/A | A/T | 0 | - | LGR6 | 1930 |
| 26 | 2034466 | T/A | Y/F | 0.01 | - | KLHDC8A | 1017 |
| 26 | 4962537 | G/A | R/W | 0 | rs316132093 | TFEB | 1092 |
| 27 | 2933980 | G/T | Q/H | 0 | - | KANSL1 | 2540 |
| 27 | 3866164 | C/T | R/H | 0.04 | - | CDK5RAP3 | 1436 |
| 27 | 4450724 | G/T | P/T | 0.01 | - | TNS4 | 1807 |
| 27 | 4824923 | G/T | A/D | 0 | - | DHX58 | 1016 |
| 28 | 754586 | C/T | P/S | 0.03 | - | RANBP3 | 121 |
| 28 | 994750 | C/T | V/M | 0 | rs317975601 | TJP3 | 2283 |
| 28 | 1129584 | C/T | R/H | 0 | - | [novel gene](http://www.ensembl.org/Gallus_gallus/Gene/Summary?g=ENSGALG00000012984&db=core) | 4655 |
| 28 | 1137335 | C/T | R/Q | 0 | - | [novel gene](http://www.ensembl.org/Gallus_gallus/Gene/Summary?g=ENSGALG00000012984&db=core) | 758 |
| 28 | 2494469 | C/T | V/M | 0.05 | - | LRG1 | 256 |
| 28 | 2539547 | C/T | R/H | 0.02 | - | ANKRD24 | 2160 |
| 28 | 2539566 | C/T | V/M | 0 | rs16210669 | ANKRD24 | 2141 |
| 28 | 3104255 | T/G | V/G | 0.02 | - | APC2 | 41 |
| 28 | 3821549 | G/C | R/G | 0.01 | rs16212208 | C19orf45 | 229 |
| Z | 2114531 | G/T | S/Y | 0.03 | - | [novel gene](http://www.ensembl.org/Gallus_gallus/Gene/Summary?g=ENSGALG00000001763&db=core) | 696 |

Supplementary Table S3.

| **Chr.** |  |  |  | **GBS** | | **60K Illum.** | | **600K Aff.** | | **GBS** | **60K** | **600K** |
| --- | --- | --- | --- | --- | --- | --- | --- | --- | --- | --- | --- | --- |
|  | **Size (Mbp)** | **%** | **Genes** | **SNPs** | **%** | **SNPs** | **%** | **SNPs** | **%** | **Density(SNP/Mbp)** | | |
| chr1 | 195.28 | 19.47 | 2,768 | 7854 | 10.02 | 7792 | 15.18 | 106607 | 17.24 | 40 | 40 | 546 |
| chr2 | 148.81 | 14.84 | 1,926 | 4776 | 6.09 | 5794 | 11.28 | 66908 | 10.82 | 32 | 39 | 450 |
| chr3 | 110.45 | 11.01 | 1,549 | 4204 | 5.36 | 4464 | 8.69 | 59738 | 9.66 | 38 | 40 | 541 |
| chr4 | 90.22 | 8.99 | 1,475 | 4636 | 5.91 | 3641 | 7.09 | 45291 | 7.32 | 51 | 40 | 502 |
| chr5 | 59.58 | 5.94 | 1,191 | 3597 | 4.59 | 2422 | 4.72 | 33047 | 5.34 | 60 | 41 | 555 |
| chrZ | 82.36 | 8.21 | 1,146 | 1194 | 1.52 | 2069 | 4.03 | 26833 | 4.34 | 14 | 25 | 326 |
| chr6 | 34.95 | 3.48 | 640 | 2519 | 3.21 | 1916 | 3.73 | 23295 | 3.77 | 72 | 55 | 666 |
| chr7 | 36.25 | 3.61 | 653 | 2356 | 3.01 | 1991 | 3.88 | 22790 | 3.69 | 65 | 55 | 629 |
| chr8 | 28.77 | 2.87 | 633 | 2288 | 2.92 | 1593 | 3.10 | 18685 | 3.02 | 80 | 55 | 650 |
| chr9 | 23.44 | 2.34 | 532 | 2790 | 3.56 | 1343 | 2.62 | 19590 | 3.17 | 119 | 57 | 836 |
| chr10 | 19.91 | 1.99 | 515 | 2884 | 3.68 | 1472 | 2.87 | 20756 | 3.36 | 145 | 74 | 1042 |
| chr11 | 19.40 | 1.93 | 442 | 2232 | 2.85 | 1418 | 2.76 | 15187 | 2.46 | 115 | 73 | 783 |
| chr12 | 19.90 | 1.98 | 407 | 2675 | 3.41 | 1526 | 2.97 | 15939 | 2.58 | 134 | 77 | 801 |
| chr13 | 17.76 | 1.77 | 441 | 3296 | 4.20 | 1293 | 2.52 | 12323 | 1.99 | 186 | 73 | 694 |
| chr14 | 15.16 | 1.51 | 487 | 3300 | 4.21 | 1194 | 2.33 | 14893 | 2.41 | 218 | 79 | 982 |
| chr15 | 12.66 | 1.26 | 430 | 2546 | 3.25 | 1231 | 2.40 | 12013 | 1.94 | 201 | 97 | 949 |
| chr16 | 0.54 | 0.05 | 86 | 152 | 0.19 | 28 | 0.05 | 1048 | 0.17 | 284 | 52 | 1958 |
| chr17 | 10.45 | 1.04 | 376 | 2922 | 3.73 | 1034 | 2.01 | 10812 | 1.75 | 280 | 99 | 1034 |
| chr18 | 11.22 | 1.12 | 377 | 3201 | 4.08 | 1016 | 1.98 | 11925 | 1.93 | 285 | 91 | 1063 |
| chr19 | 9.98 | 1.00 | 383 | 2493 | 3.18 | 1008 | 1.96 | 11047 | 1.79 | 250 | 101 | 1107 |
| chr20 | 14.30 | 1.43 | 443 | 2799 | 3.57 | 1731 | 3.37 | 10968 | 1.77 | 196 | 121 | 767 |
| chr21 | 6.80 | 0.68 | 286 | 1403 | 1.79 | 938 | 1.83 | 10440 | 1.69 | 206 | 138 | 1535 |
| chr22 | 4.08 | 0.41 | 147 | 627 | 0.80 | 359 | 0.70 | 5101 | 0.82 | 154 | 88 | 1250 |
| chr23 | 5.72 | 0.57 | 268 | 2215 | 2.83 | 754 | 1.47 | 7910 | 1.28 | 387 | 132 | 1382 |
| chr24 | 6.32 | 0.63 | 214 | 1966 | 2.51 | 873 | 1.70 | 8888 | 1.44 | 311 | 138 | 1406 |
| chr25 | 2.19 | 0.22 | 255 | 826 | 1.05 | 267 | 0.52 | 3877 | 0.63 | 377 | 122 | 1769 |
| chr26 | 5.33 | 0.53 | 279 | 2623 | 3.35 | 790 | 1.54 | 7816 | 1.26 | 492 | 148 | 1466 |
| chr27 | 5.21 | 0.52 | 334 | 2026 | 2.58 | 621 | 1.21 | 7087 | 1.15 | 389 | 119 | 1360 |
| chr28 | 4.74 | 0.47 | 299 | 1974 | 2.52 | 765 | 1.49 | 7051 | 1.14 | 416 | 161 | 1487 |
| chr32 | 0.00 | 0.00 |  | 16 | 0.02 | 0 | 0.00 | 0 | 0.00 | 15564 | 0 | 0 |
| chrW | 1.25 | 0.12 | 13 | 9 | 0.01 | 0 | 0.00 | 14 | 0.00 | 7 | 0 | 11 |
| chrLGE | |  | 61 | 0 | 0.00 | 0 | 0.00 | 261 | 0.04 |  |  |  |
| chrLGE64 | |  | 50 | 0 | 0.00 | 0 | 0.00 | 168 | 0.03 |  |  |  |
| **Total** | 1,003 | 100 |  |  | 51,343 | 100 | 618,308 | 100 |  |  |  |  |

Supplementary Table S4

| **Variants** | **CornellGBS** | | **60K Illumina** | | **600K Affymetrix** | |
| --- | --- | --- | --- | --- | --- | --- |
|  | **Total no.** | **%** | **Total no.** | **%** | **Total no.** | **%** |
| *All variants* | 78399 | 100 | 51335 | 100 | 618308 | 100 |
| Intronic | 28181 | 35.95 | 18813 | 36.65 | 238071 | 38.50 |
| Intergenic | 22116 | 28.21 | 22423 | 43.68 | 8921 | 1.44 |
| Exonic | 2590 | 3.30 | 1338 | 2.61 | 21694 | 3.51 |
| Splicing | 256 | 0.33 | 166 | 0.32 | 1504 | 0.24 |
| ncRNA | 6 | 0.01 | 2 | 0.00 | 6 | 0.00 |
| 5'-UTR | 268 | 0.34 | 89 | 0.17 | 890 | 0.14 |
| 3'-UTR | 1328 | 1.69 | 650 | 1.27 | 6948 | 1.12 |
| Usptream (1kb) | 11516 | 14.69 | 4078 | 7.94 | 46849 | 7.58 |
| Downstream (1kb) | 12306 | 15.70 | 3870 | 7.54 | 45002 | 7.28 |
| *Exonic* |  |  |  |  |  |  |
| Synonymous SNP | 1671 | 64.52 | 379 | 28.33 | 12671 | 58.41 |
| Non-synonymous SNP | 907 | 35.02 | 888 | 66.37 | 8921 | 41.12 |
| Startlost SNP | 5 | 0.19 | 5 | 0.37 | 5 | 0.02 |
| Stopgain SNP | 3 | 0.12 | 3 | 0.22 | 94 | 0.43 |
| Stoplost SNP | 4 | 0.15 | 63 | 4.71 | 3 | 0.01 |

**3. Supplementary data**

Supplementary Data S1. CornellGBS workflow

**Preparation of Adapters**

Resuspend the lyophilized oligos in the necessary volume of TE to obtain a concentration of 200 µM

*Adapters Annealing*

In tubes of 0.2 mL or plates, adapters F and R were added (forward and reverse, respectively) as follows:

| **Product** | **Volume** |
| --- | --- |
| primer F | 25 µL |
| primer R | 25 µL |
| TE | 50 µL |
| **Total** | 100 µL (50 mM) |

In the thermal cycler: 95 ^o^C per 2´, 25 ^o^C per 30´, 4 ^o^C per ∞ (ramp for 25 ^o^C - 0.1 ^o^C/seconds)

*First dilution*

Raw stock

| **Product** | **Volume** |
| --- | --- |
| Barcode annealed | 6 µL |
| TE 1X | 994 µL |
| **Total** | 1,000 µL |

vortex and spin

Quantification of adapters using Qubit®Fluorometric Quantification

Commum adapter: 107.3 ng/µL

Barcode adapters: ~6.5 ng/µL

*Second dilution*

Concentrated stock

Mix "barcode" with "common"adapters for a concentrated stock (the common adapter is the same on each linkage)

The composition of each 0.2 tube or well of the plate was:

| **Product** | **Amount** |
| --- | --- |
| barcode adapter | 300 ng |
| commum adapter | 300 ng |
| TE 1X | ? µL |
| **Total** | 200 µL (3 ng/µL) |

Vortex and spin

**Stock in 3 ng/µL**

*Third dilution*

Work stock

The concentrated stock was diluted 1:4 (stock:water) for a final concentration of 0.6 ng/µL.

In this case, the amount of the adapters must be optimized according to the genome species investigated.

**Lab Workflow**

To quantify the DNA in fluorometer (Qubit®Fluorometric Quantification, e.g) samples were diluted to 50 ng/µL (minimum required is 100 ng).

*Cleavage reaction:*

| **Reagent** | **1X** |
| --- | --- |
| DNA (100 ng) | 2 µL |
| NEB buffer3 | 3 µL |
| Pst1 (10U/µL) | 0.2 µL |
| UP Water | 24.7 µL |
| **Total** | 30 µL |

vortex and spin

Incubation was performed at 37 °C for 2 hours and 85 °C for 20' (these correspond to activity and deactivation temperatures of *PstI* restriciton enzyme, respectively)

Verification of DNA cleavage was performed in 1% agarose gel using the Low DNA Mass Ladder (Invitrogen™) to verify the size of the DNA smear (see Supplementary Fig. S1 online).

Samples were then dryied.

*Binding Reaction*

6 µL of working solutions adapdator were added (this varies according to the optimization performed for each species) to dried DNA from the previos step. Incubation was performed for 30 min at room temperature (RT) for optimal resuspension of DNA (using a plate shaker).

Binding mix was added to each sample as follow:

| **Reagent** | **1X** |
| --- | --- |
| 10XT4DNA Buffer | 2 µL |
| T4 DNA Ligase (400U) | 1 µL |
| UP Water | 21 µL |
| **Total** | **24 µL** |
| Adapter+DNA (one plate) | 6 µL |
| **Total** | **30 µL** |

vortex and spin

After that, the plate was incubated at 22 °C for 2 hours and 65 °C for 30'

*Pool Preparation*

Two pools of 24 samples each (to be loaded in one Illumina flowcell lane) were prepared as follows:

10 uL of the “bound” material of each sample, totaling 240 µL plus 5 volumes of Binding Buffer PB was purified by QIAquick PCR Purification Kit (QUIAGEN), totaling 1,440 µL (240 µL of DNA+1,200 µL of PB) per pool.

Vortex, spin and purification steps were performed according to manufactured protocol.

The content was ressuspended in 30 µL of Buffer EB (QIAquick protocol).

*Polymerase Chain Reaction (PCR)*

The PCR was performed as follow:

| **Reagent** | **1X** |
| --- | --- |
| Buffer 10X | 5 µL |
| MgCl_2_(50mM) | 2 µL |
| dNTP | 1.5 µL |
| Taq | 0.25 µL |
| Primer A (20uM) | 1.25 µL |
| Primer B (20uM) | 1.25 µL |
| UP Water | 23.75 µL |
| **Total** | 35 µL |
| PoolDNA | 15 µL |
| Total | 50µL |

Program in the thermal cycler

Step 1- 72 ^o^C per 5´

Step 2 - 98 ^o^C per 30´´

Step 3 - 98 ^o^C per 10´´

Step 4 - 65 ^o^C per 30´´

Step 5 - 72 ^o^C per 30´´

Step 6 – go to step 3 and repeat 18 times

Step 7 - 72 ^o^C per 5´

Step 8 - 4 ^o^C ∞

A 1% agarose gel run was performed with the PCR product and a ladder to verify the fragments size that should be concentrated between 200 and 500 bp (see Supplementary Fig. S2 online).

*PCR purification*

The purification of the PCR for sequencing was carried out through Agencourt®XP AMPURE manufactured protocol using 1.5 µL of Agencourt AMPure XP reagent to each 1 µL of PCR products. We suggest purifying each PCR product into two aliquots of 20 µL.

A 1% agarose gel run was performed with the purified library pool and the ladder to verify the fragments size range obtained afer the PCR purification step. This gel verifyies the elimination of the ~50 bp fragment corresponding to the unlinked primers) (see Supplementary Fig. S2 online).

Each library was quantified by qPCR using the *KAPA Library Quantification Kit* (KAPA Biosystems).
